# Contrast-Enhanced Mammographic Features of In Situ and Invasive Ductal Carcinoma Manifesting Microcalcifications Only: Help to Predict Underestimation?

**DOI:** 10.3390/cancers13174371

**Published:** 2021-08-30

**Authors:** Yun-Chung Cheung, Kueian Chen, Chi-Chang Yu, Shir-Hwa Ueng, Chia-Wei Li, Shin-Cheh Chen

**Affiliations:** 1 Department of Medical Imaging and Intervention, Chang Gung Memorial Hospital, Medical College of Chang Gung University, 5 Fuxing St., Guishan, Taoyuan 333, Taiwan; chenkueian@gmail.com; 2 Division of Breast Surgery, Department of Surgery, Chang Gung Memorial Hospital, Medical College of Chang Gung University, 5 Fuxing St., Guishan, Taoyuan 333, Taiwan; kenneth0609@cgmh.org.tw (C.-C.Y.); chensc@cgmh.org.tw (S.-C.C.); 3 Department of Pathology, Chang Gung Memorial Hospital, Medical College of Chang Gung University, 5 Fuxing St., Guishan, Taoyuan 333, Taiwan; shu922@cgmh.org.tw; 4 Research Group, GE Health Care, Taipei 11031, Taiwan; chiawei.lee@gmail.com

**Keywords:** breast cancer, contrast-enhanced spectral mammography, microcalcification, breast biopsy, ductal carcinoma in situ

## Abstract

**Simple Summary:**

Mammography frequently detects the early breast cancers manifesting microcalcifications only. By means of mammographically guided vacuum-assisted core needle biopsy, noninvasive ductal carcinoma in situ (DCIS) is often diagnosed. Unfortunately, invasive components are occasionally embedded within the noninvasive cancer bed, which will alter the preoperative planning. Whether or not to perform single-step sentinel lymph node sampling is an area of controversy. In order to minimize the overtreatment or undertreatment, we retrospectively reviewed the enhanced features of DCIS and invasive ductal carcinomas (IDCs) on contrast-enhanced spectral mammograms (CESMs). The CESM is a modern technique enabling the provision of conventional mammograms and contrast-enhanced images. The results of our study revealed low DCIS upgrade of the unenhanced DCIS. Otherwise, DCIS tended to appear as nonmass and pure ground glass enhancements, and IDC tended to appear as mass and unpurified solid enhancement. The enhanced features allow distinguishing DCIS and IDC.

**Abstract:**

Background: The contrast-enhanced mammographic features of ductal carcinoma in situ (DCIS) and invasive ductal carcinoma (IDC) manifesting microcalcifications only on mammograms were evaluated to determine whether they could predict IDC underestimation. Methods: We reviewed patients who underwent mammography-guided biopsy on suspicious breast microcalcifications only and received contrast-enhanced spectral mammography (CESM) within 2 weeks before the biopsy. Those patients who were proven to have cancers (DCIS or IDC) by biopsy and subsequently had surgical treatment in our hospital were included for analysis. The presence or absence, size, morphology and texture of enhancement on contrast-enhanced spectral mammography were reviewed by consensus of two radiologists. Results: A total of 49 patients were included for analysis. Forty patients (81.6%) showed enhancement, including 18 (45%) DCIS and 22 (55%) IDC patients. All nine unenhanced cancers were pure DCIS. Pure DCIS showed 72.22% nonmass enhancement and 83.33% pure ground glass enhancement. IDC showed more mass (72.2% vs. 27.8%) and solid enhancements (83.33% vs. 16.67%). The cancer and texture of enhancement were significantly different between pure DCIS and IDC, with moderate diagnostic performance for the former (*p*-value < 0.01, AUC = 0.66, sensitivity = 93%, specificity = 39%) and the latter (*p*-value < 0.01, AUC = 0.74, sensitivity = 65%, specificity = 83%). Otherwise, pure DCIS showed a significant difference in enhanced texture compared with upgraded IDC and IDC (*p* = 0.0226 and 0.0018, respectively). Conclusions: Nonmass and pure ground glass enhancements were closely related to pure DCIS, and cases showing mass and unpurified solid enhancements should be suspected as IDC. Unenhanced DCIS with microcalcifications only has a low DCIS upgrade rate. The CESM-enhanced features could feasibly predict IDC underestimation.

## 1. Introduction

The manifestation of suspicious microcalcifications only on mammography is indicative of early cancers. Approximately 20–25% of these patients are diagnosed with malignancy by mammography-guided needle biopsy [1,2,3]. Most of the cases are noninvasive ductal carcinoma in situ (DCIS); however, unfortunately, invasive components occasionally embed within the noninvasive cancer bed, which will alter preoperative planning. Regardless of DCIS or invasive ductal carcinoma (IDC), it is universally agreed that management with subsequent surgical treatment either with conservative or total mastectomy should be recommended [4,5,6]. However, being able to predict the underestimation of biopsy-proven DCIS will facilitate preoperative planning, in which it is not essentially advised to perform sentinel lymph node sampling for cases of pure DCIS [7]. Obviation of the supplementary performance of sentinel lymph node biopsy will provide benefits, including shortening the operative time, avoiding unnecessary exposure to radiation doses or minimizing the potential complications of lymph node resection.

Mammography-guided vacuum-assisted core needle biopsy is commonly used to histologically diagnose the etiology of suspicious microcalcifications. The performance has improved from spring-loaded biopsy to vacuum-assisted large needle biopsy, which approximates surgical biopsy [8]. However, IDC underestimation by needle biopsy occasionally occurs in clinical practice. A meta-analysis of 7350 cases of biopsy-diagnosed DCIS including masses or microcalcifications from 52 studies reported a 30.3% underestimation rate for 14-gauge core needles and an 18.9% underestimation rate for 11-gauge vacuum-assisted needle biopsy [9]. Even when using a large-bore 7-gauge biopsy needle, the upgrade rate of DCIS to IDC is still 15.38% [10].

Multiple imaging modalities, including conventional mammography and sonography, have been used in an attempt to predict IDC underestimation [11,12,13,14]. In this study, we investigate a new imaging technique, contrast-enhanced spectral mammography (CESM), which can provide conventional low-energy mammograms (LMs) and recombined enhanced images (REIs) from the same session of a single compressed position. With this exclusive benefit, concerning enhancements on REIs and suspicions on conventional mammograms can be easily correlated and assessed. In the past, research mainly focused on the diagnostic performance of CESM. In this retrospective study, we compared the enhanced features of biopsy-diagnosed breast cancers, including DCIS and IDC, manifesting mammographic microcalcifications only on conventional mammograms to analyze the feasibility of predicting IDC underestimation preoperatively.

## 2. Materials and Methods

### 2.1. Patient Selection

With approval from the IRB of our hospital, we reviewed patients who underwent mammography-guided biopsy, had suspicious breast microcalcifications only and underwent CESM within 2 weeks before the biopsy. Patients who were proven to have cancers (including DCIS or IDC) by biopsy and subsequently received surgical treatment in our hospital were included for analysis.

Patients with (1) abnormal renal function (abnormal serum creatinine > 1.0 mg/dL and glomerular filtration rate 60 mL/min/1.73 m^2^), (2) pregnancy, (3) lactation, (4) history of allergy to iodized contrast medium, (5) past history of breast cancer or surgery before biopsy or (6) systemic disease such as hyperthyroidism were clinically excluded from receiving CESM examination. The CESM examination method and potential side effects of contrast medium were fully explained to those who underwent CESM. Finally, all the patients signed agreements to participate in this study according to our hospital regulations.

### 2.2. Image Analysis

The CESM (Senographe Essential or Pristina CESM; GE Healthcare, Buc, France) examination was standardized and performed with intermittent exposure (approximately 2-second intervals) to low and high energy (below and above the k-edge of iodine: 33.2 keV) during a single breast-compressed position. The image acquisitions were routinely obtained in the sequence of craniocaudal (CC) and mediolateral oblique (MLO) views of the bilateral breasts within 2–6 min after the start of a single-bolus injection of nonionic contrast medium (Omnipaque 350 mg I/mL; GE Healthcare, Dublin, Ireland) at a rate of 3 mL/s for a total dose of 1.5 mL/kg body weight via an intravenous catheter that was inserted into the forearm prior to the examination.

**Figure 1:**
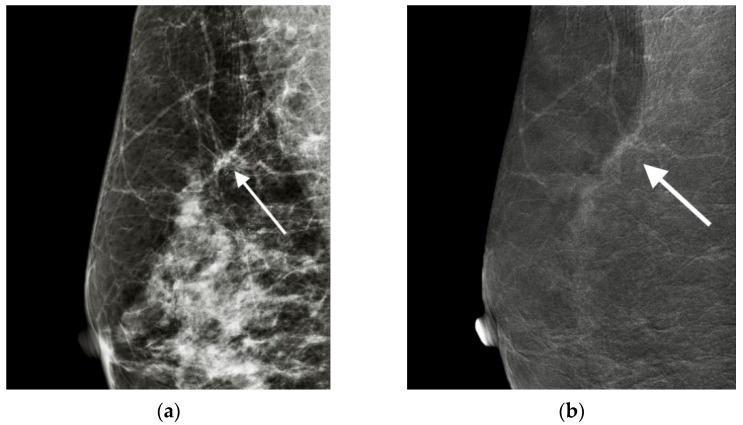
Nonmass with pure ground glass enhancement: In a 59-year-old woman, the MLO view of CESM showed the group of linear and pleomorphous microcalcifications in the upper region of the right breast (arrow) on LM (**a**), revealing nonmass with pure ground glass enhancement (arrow) on REI (**b**). Mammographically guided core needle biopsy and surgery concordantly diagnosed DCIS.

**Figure 2:**
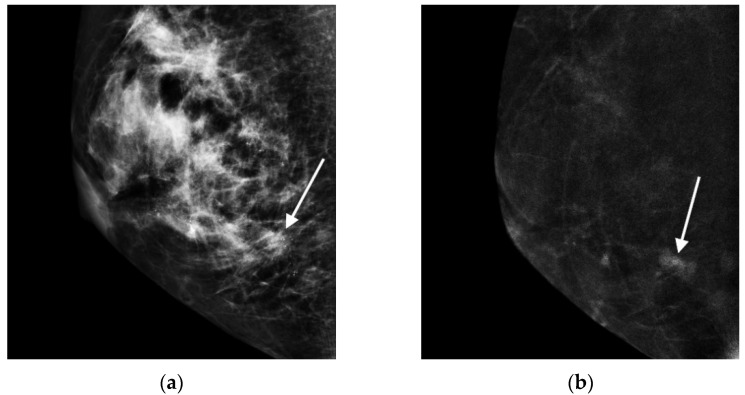
Mass with unpurified ground glass enhancement: In a 57-year-old woman, the biopsied microcalcifications were a group of pleomorphous microcalcifications (arrow) in the lower region of the right breast on LM in MLO view (**a**). REI revealed an associated irregular mass with ground glass and solid enhancement (arrow) (**b**). Finally, it was surgically proven to be IDC.

All the CESM data, including the LMs and REIs, were reviewed, and the results were determined by consensus of two radiologists (8 years and 3 years CESM experiences). The locations of the biopsied microcalcifications were first identified on the LM and then evaluated for the enhancement features on the REI. The presence or absence of enhancement was first recorded, and enhancement features, including size, morphology and texture, were evaluated. The size of cancer enhancement was measured in the greatest diameter in either the CC or MLO view. The enhancement morphology was classified as nonmass (clump appearance without a bulging outline) or mass (shaped appearance with a bulging outline). The textures of enhancement consisted of pure ground glass (transparent to underlying) (Figure 1) and unpurified ground glass (with a nontransparent solid part) (Figure 2). For the reason of unmeasurable enhancement intensity on REI, we used the terms of ‘ground glass’ and ‘solid’ appearances to distinguish the texture of ‘weaker’ and ‘stronger’ enhancement relevant to the transparency to the underlying even though they were infrequently used as enhancement descriptors.

### 2.3. Statistical Analysis

We used the Mann–Whitney U test for statistical analysis of the significance of enhanced features between DCIS and IDC. The significance was then evaluated by univariable logistic regression for diagnostic performance. All statistical analyses were performed with SPSS software version 20.0 (SPSS, Chicago, IL, USA). We also used the Wilcoxon rank-sum test to analyze the significant differences among the three groups of pure DCIS, upgraded IDC and IDC. A *p*-value of < 0.05 was set to indicate statistical significance.

## 3. Results

### 3.1. Patient Characteristics

From 2015 to 2020, 56 patients with breast cancer (44 DCIS and 12 IDC) diagnosed by stereotactically or tomographically guided vacuum-assisted core needle biopsy were identified from our records. However, only 49 breast cancer patients (39 DCIS and 10 IDC) were enrolled for analysis after excluding seven patients without subsequent operation in our hospital. Of the 39 biopsy-diagnosed DCIS cases, 12 (30.77%) were surgically/histologically upgraded to IDC. Finally, 27 patients with pure DCIS and 22 patients with IDC were compared in this study.

The average ages of patients with pure DCIS and IDC were approximately the same (53.9 years vs. 51.4 years), ranging from 44 to 75 years and 30 to 62 years, respectively. The morphologies of biopsied microcalcifications on mammography were recorded as amorphous in 15 cases, pleomorphic in 24, linear in 7 and casting in 3; the distributions were 27 in group, 7 in region, 13 in segment and 2 in linear (Table 1). All the microcalcifications were finally classified into category 4 of the American College of Radiology Breast Imaging and Reporting Data System (ACR BI-RADS) after assessment and recommended for mammography-guided vacuum-assisted core needle biopsy.

### 3.2. Comparison of DCIS and IDC

In total, 40 of 49 (81.6%) microcalcifications were observed to have associated enhancement. Of the final surgically/histologically diagnosed pure DCIS and IDC cases (27 and 22 cases, respectively), 18 (66.6%) pure DCIS and all IDC (100%) cases showed enhancement. The presence of enhancement did not show a significant difference between pure DCIS (45%) and IDC (55%). However, all nine unenhanced microcalcifications were finally proven to be pure DCIS. The average cancer sizes measured on REI were 1.46 cm (0 to 6.5 cm) for pure DCIS and 2.93 cm (0.8 to 8.6 cm) for IDC, without a significant difference between the two groups.

The enhancement features of pure DCIS and IDC are listed in Table 2. Pure DCIS showed 72.22% nonmass enhancement and 83.33% pure ground glass enhancement. IDC had similar percentages of cases with mass and nonmass morphologies; otherwise, predominantly 68.18% of cases showed solid enhancement. However, the features of both pure DCIS and IDC could overlap. Regarding the mass and texture enhancements, IDC showed more enhanced masses (72.2% vs. 27.8%) and solid enhancement (83.33% vs. 16.67%) than pure DCIS.

The presence or absence of enhancement and the enhanced texture were significantly different between the two groups. Univariate logistic regression further showed moderate diagnostic performance in the former (*p*-value < 0.01, AUC = 0.66, sensitivity = 93%, specificity = 39%) and in the latter (*p*-value < 0.01, AUC = 0.74, sensitivity = 65%, specificity = 83%).

### 3.3. Statistical Analysis of the Pure DCIS, Upgraded IDC and IDC Groups

**Figure 3:**
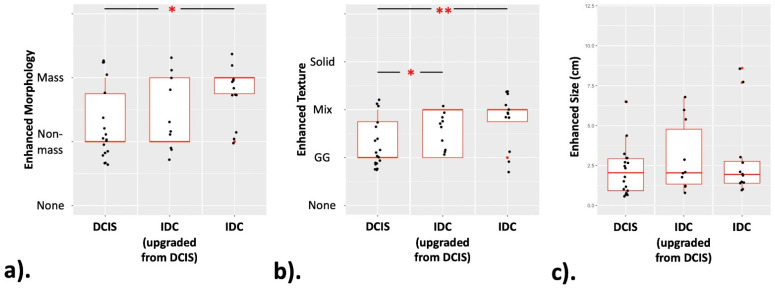
The box plots of three lesion groups. These demonstrate the distribution of (**a**) enhanced morphology, (**b**) enhanced texture and (**c**) enhanced size, among three groups. (*: *p* < 0.05; **: *p* < 0.01).

The enhancement features of pure DCIS, upgraded IDC and IDC are listed in Table 3. The Wilcoxon rank-sum test was used to analyze the significant differences among three groups of pure DCIS (27 cases), upgraded IDC (12 cases) and IDC (10 cases). The pure DCIS group showed a significant difference in morphology compared with the IDC group (*p* = 0.0134) and in enhanced texture compared with the upgraded IDC and IDC groups (*p* = 0.0226 and 0.0018, respectively). These results are shown in Figure 3.

## 4. Discussion

The new modality we investigated in this study, CESM, is a novel mammography-based imaging examination that was approved for clinical use in 2011 [15]. Utilizing the different attenuation coefficients of iodine and glandular tissues under low- and high-energy exposures, computer software can recombine the low- and high-energy images to highlight the presence of iodine accumulation after eliminating the breast tissue background. The enhanced lesions indicate possible pathogenic lesions. The sensitivity of CESM ranges from 93% to 100%, and the specificity ranges from 63% to 88%, showing significant improvement compared to full-field digital mammography [16,17,18]. Particularly for dense breasts, CESM beneficially increased the cancer sensitivity and specificity by approximately 22% and 16%, respectively [19].

Although mammography is sensitive for microcalcification detection, the cancer probability ranges widely among the various morphologies or distributions of microcalcifications on mammograms, from 2% to 95% in accordance with ACR BI-RADS 4 [3]. By using an enhancing technique, advanced contrast-enhanced magnetic resonance imaging (CE-MRI) can detect the associated enhancement of glandular tissues adjacent to suspicious microcalcifications. A meta-analysis analyzed 1843 lesions from 20 studies and reported a pooled sensitivity and specificity of 92% and 82%, respectively, for BI-RADS 4 microcalcifications [20]. CESM has an approximate performance to CE-MRI, with 88.89% sensitivity, 86.56% specificity, 72.72% positive predictive value and 95.08% negative predictive value from a screening population [21]. Compared to CE-MRI, CESM more easily correlates the microcalcifications on the LM to surrounding glandular enhancement on the REI in the same session of positioning. Another test was designed to have radiologists read the LM first and then read the LM with the REI a day later. The sensitivity, specificity, positive predictive value and negative predictive value of CESM were mildly improved from LM, from 93.8% to 96.8%, from 36.6% to 34.1%, from 54% to 54% and from 88.2% to 92.2%, respectively [22]. Conversely, the enhancement information from REI did not seem to affect the decision in terms of the bias of personal mammographic knowledge on microcalcifications. Another investigation recently reported a significantly higher predictive positive value and lower misdiagnosis rate when using REIs than when using LMs alone, and a machine learning model could significantly improve the diagnosis of both low-risk (AUC 0.77 using LMs alone to 0.9 with REIs) and high-risk (AUC 0.71 using LMs alone to 0.86 with REIs) groups of microcalcifications [23]. The additional REI seemed helpful in assessing suspicious microcalcifications.

For the management of suspicious microcalcifications, mammography-guided vacuum-assisted needle biopsy, either with stereotactic or tomosynthesis techniques, is clinically used as a standard procedure to obtain calcified specimens for pathologic diagnosis. Once microcalcifications are diagnosed as cancer, subsequent surgery should be performed to remove the residual cancer cells. However, whether sentinel lymph node sampling is performed depends on the actual nature of pure DCIS or IDC.

Due to the limited amount of specimens obtained by needle biopsy, there is a risk of underestimation. In this series, the IDC underestimation rate was 30.77% (12 of 39 biopsy-diagnosed DCIS upgraded to IDC after surgery). To gain more knowledge for preoperative prediction, we analyzed information on cancer enhancement from CESM, including the presence or absence of enhancement and the enhancement extent, morphology and texture of cancers. From our results, all the unenhanced microcalcifications were DCIS, and all the IDC cases showed enhancement. The incidence of DCIS upgrades seemed rare in cases of unenhanced needle-diagnosed DCIS manifesting only microcalcifications. This result was speculated to be due to the silent or less aggressive behavior of DCIS.

The preoperative measurement of cancer extent to predict whether a coexisting invasive component exists may serve as a guideline for surgeons to consider sentinel lymph node sampling [7]. Larger cancers tend to have a greater chance of having invasive components. The cut-off sizes of DCIS underestimation varied, ranging from 1.1 to 4 cm [24,25,26], which was unfortunately variable in different patient collections. In our CESM study, the average size of IDC (2.93 cm) was larger than that of DCIS (1.46 cm), but the difference was statistically nonsignificant.

In terms of morphology, using mass or nonmass descriptions to characterize a cancer is theoretically related to the growth pattern of cancer. The mass morphology is defined as a localized lesion with a bulging outline configuration, and the nonmass morphology is an infiltrating pattern with a clump appearance. In our study, the incidence of nonmass enhancement was approximately 59.1% for pure DCIS and 40.9% for IDC. More IDC cases presented mass enhancement than DCIS cases (72.2% vs. 27.8%). In fact, similar results from previous CE-MRI studies have shown that the presence of a mass lesion was a preoperative predictor of DCIS with an invasive component [27].

Stronger enhancement secondary to richer neovascularity indicates the rapid or aggressive behavior of a cancer. Such enhancement that can obscure the underlying tissue is herein described as solid enhancement. In this study, we used the term ‘ground glass’ to describe the texture of mild enhancement with transparent visualization to the background; otherwise, in the cases of unpurified texture, we indicate the presence of nontransparent solid enhancement. Unpurified enhancement is predominantly observed in IDC compared to DCIS, accounting for 83.3% vs. 16.7% of cases.

Prospectively, CESM can improve the performance of mammographically guided biopsy. In cases of suspicious microcalcifications, only the specimen radiography enables to document the retrieval of microcalcifications for diagnosis. However, it cannot predict the IDC underestimation. The underestimation absolutely depends on microscopic findings. The biopsy target and whether invasive parts are obtained for microscopic evaluation are key to avoiding underestimation. The obtained microcalcifications in the specimens are not equivalent in terms of the presence of invasive elements. Other than for the purpose of diagnosis, enhancement features such as mass and solid enhancements on CESM can provide suggestive sites for biopsy that may improve the diagnosis of invasion. The usefulness of CESM-guided biopsy should be proven in the future.

There are several limitations in this study: (1) The case number was small and the patients were not consecutive. CESM was not a compulsory examination prior to biopsy in the current clinical practice, and certain patients hesitated to undergo contrast medium injection due to the potential side effects. (2) The CESM features of pure DCIS and IDC were consistently analyzed by consensus of two radiologists. Although interobserver bias might be present, the interpretation of pure DCIS and IDC was basically achieved. Prospective or blind study of interobserver agreement should be designed in the future.

## 5. Conclusions

Our outcomes showed nonmass and pure ground glass enhancements were closely related to pure DCIS, and cases showing mass and unpurified solid enhancements should be suspected as IDC. Unenhanced pure DCIS manifesting microcalcifications only had a low DCIS upgrade rate. CESM features can feasibly predict IDC underestimation.

## Figures and Tables

**Table 1:** Characteristics of patients and microcalcifications.

	DCIS (27)	IDC (22)
Ages (years)	53.9 (44–75)	51.4 (30–62)
Side		
Right	14	10
Left	13	12
Calcification Morphology		
Amorphous	7	8
Pleomorphous	13	11
Cast	2	1
Linear	5	2
Calcification Distribution		
Group	19	8
Region	2	5
Segment	4	9
Linear	2	0

**Table 2:** Comparison of CESM enhanced features of pure DCIS and IDC.

	DCIS (27)	IDC (22)	*p*-Valve
Enhancement			<0.01
Presence	18 (66.67%)	22 (100%)	
Absence	9 (33.33%)	0 (0%)	
Average Size (cm)	1.46 (0–6.5)	2.93 (0.8–8.6)	0.26
Enhanced Cancers	DCIS (18)	IDC (22)	
Enhanced Morphology			0.05
Presence of Mass	5 (27.78%)	13 (59.1%)	
Nonmass	13 (72.22%)	9 (40.9%)	
Enhancement Texture			<0.01
Pure Ground Glass	15 (83.33%)	7 (31.82%)	
Unpurified Ground Glass	3 (16.67%)	15 (68.18%)	

Statistic analyzed by Mann–Whitney U test with significant *p*-value < 0.05.

**Table 3:** Comparison of CESM enhanced features of pure DCIS, upgraded IDC and IDC.

	DCIS (27)	Upgraded IDC (12)	IDC (10)
Enhancement			
Presence			
Yes	18 (66.67%)	12 (100%)	10 (100%)
No	9 (33.33%)	0	0
Average Size (cm)	1.46 (0–6.5)	3.02 (0.8–8.6)	2.86 (1–7.7)
Enhanced Cancers	18	12	10
Enhanced Morphology			
Presence of Mass	5 (27.78%)	5 (41.67%)	8 (80%)
Nonmass	13 (72.22)	7 (58.33%)	2 (20%)
Enhancement Texture			
Pure Ground Glass	15 (83.33%)	5 (41.67%)	0
Unpurified Ground Glass	3 (16.67%)	7 (58.33%)	12 (100%)

## Data Availability

The datasets used and/or analyzed during the current study are available from the corresponding author on reasonable request.

## Abbreviations

DCIS	Contrast-medium spectral mammography
DCIS	Ductal carcinoma in situ
IDC	Invasive ductal carcinoma
LMs	Low-energy mammograms
REIs	Recombined enhanced images
CC	Craniocaudal
MLO	Mediolateral oblique
ACR	American College of Radiology
BI-RADS	Breast Imaging and Reporting Data System
CE-MRI	Contrast-enhanced magnetic resonance imaging
